# Apparently synonymous substitutions in *FGFR2* affect splicing and result in mild Crouzon syndrome

**DOI:** 10.1186/s12881-014-0095-4

**Published:** 2014-08-31

**Authors:** Aimee L Fenwick, Jacqueline AC Goos, Julia Rankin, Helen Lord, Tracy Lester, A Jeannette M Hoogeboom, Ans MW van den Ouweland, Steven A Wall, Irene MJ Mathijssen, Andrew OM Wilkie

**Affiliations:** 1 Weatherall Institute of Molecular Medicine, John Radcliffe Hospital, University of Oxford, Headington, Oxford OX3 9DS, UK; 2 Department of Plastic and Reconstructive Surgery and Hand Surgery, Erasmus MC, University Medical Center, Rotterdam, The Netherlands; 3 Clinical Genetics Department, Royal Devon and Exeter NHS Foundation Trust, Exeter, UK; 4 Genetics Laboratories, Oxford University Hospitals NHS Trust, Churchill Hospital, Oxford, UK; 5 Department of Clinical Genetics, Erasmus MC, University Medical Center, Rotterdam, The Netherlands; 6 Craniofacial Unit, Oxford University Hospitals NHS Trust, John Radcliffe Hospital, Oxford, UK

**Keywords:** Craniosynostosis, Crouzon syndrome, Expressivity, FGFR2, Penetrance, Splicing, Synonymous substitution

## Abstract

**Background:**

Mutations of *fibroblast growth factor receptor 2* (*FGFR2*) account for a higher proportion of genetic cases of craniosynostosis than any other gene, and are associated with a wide spectrum of severity of clinical problems. Many of these mutations are highly recurrent and their associated features well documented. Crouzon syndrome is typically caused by heterozygous missense mutations in the third immunoglobulin domain of *FGFR2*.

**Case presentation:**

Here we describe two families, each segregating a different, previously unreported *FGFR2* mutation of the same nucleotide, c.1083A>G and c.1083A>T, both of which encode an apparently synonymous change at the Pro361 codon. We provide experimental evidence that these mutations affect normal *FGFR2* splicing and document the clinical consequences, which include a mild Crouzon syndrome phenotype and reduced penetrance of craniosynostosis.

**Conclusions:**

These observations add to a growing list of *FGFR2* mutations that affect splicing and provide important clinical information for genetic counselling of families affected by these specific mutations.

## Background

Craniosynostosis defines the premature fusion of the cranial sutures and has an overall prevalence of 1 in 2100–2300 live births [1],[2]. Nearly one quarter of craniosynostosis has a genetic aetiology [3],[4]; there is considerable genetic heterogeneity and frequent phenotypic overlap between different syndromes. Genes encoding three members of the fibroblast growth factor receptor family (*FGFR1*, *FGFR2* and *FGFR3*) are commonly mutated in individuals with craniosynostosis. Heterozygous mutations in *FGFR2*, which are frequently recurrent, account for ~28% of genetic cases [4] and cause Crouzon [5],[6], Pfeiffer [7]–[9], Apert [10], Beare-Stevenson [11] and bent bone dysplasia [12] syndromes. All involve synostosis of the coronal and other cranial sutures, a distinctive “crouzonoid” craniofacial appearance (comprising hypertelorism, exorbitism, prominent nose, and midface hypoplasia), but differ in the presence and extent of abnormalities of the hands and feet, other skeletal manifestations and dermatological features [13],[14].

FGFR2, like the other members of the FGFR family, comprises an extracellular ligand-binding region (composed of three immunoglobulin-like domains), a single transmembrane peptide and a cytoplasmic tyrosine kinase domain. Mutually exclusive alternative splicing of exons IIIb and IIIc gives rise to epithelial and mesenchymal isoforms (FGFR2b and FGFR2c) respectively [15]. These alternative extracellular domains interact with different repertoires of fibroblast growth factors (FGFs) to regulate downstream processes such as proliferation, differentiation and cell migration [16].

Here we describe two families heterozygous for the same, previously unreported apparently synonymous variant in *FGFR2* [p.(Pro361Pro)], although caused by differing nucleotide substitutions. The mutation carriers in both families exhibit features of mild Crouzon syndrome, and a minority required craniofacial surgery. We propose that this variant is in fact pathogenic and demonstrate the generation of abnormal cDNA products resulting from incorrect splicing of exon IIIc in the mutant allele. This finding highlights the challenges posed in interpreting such synonymous variants when providing genetic counselling for affected families.

## Case presentations

### Family 1

**Figure 1:**
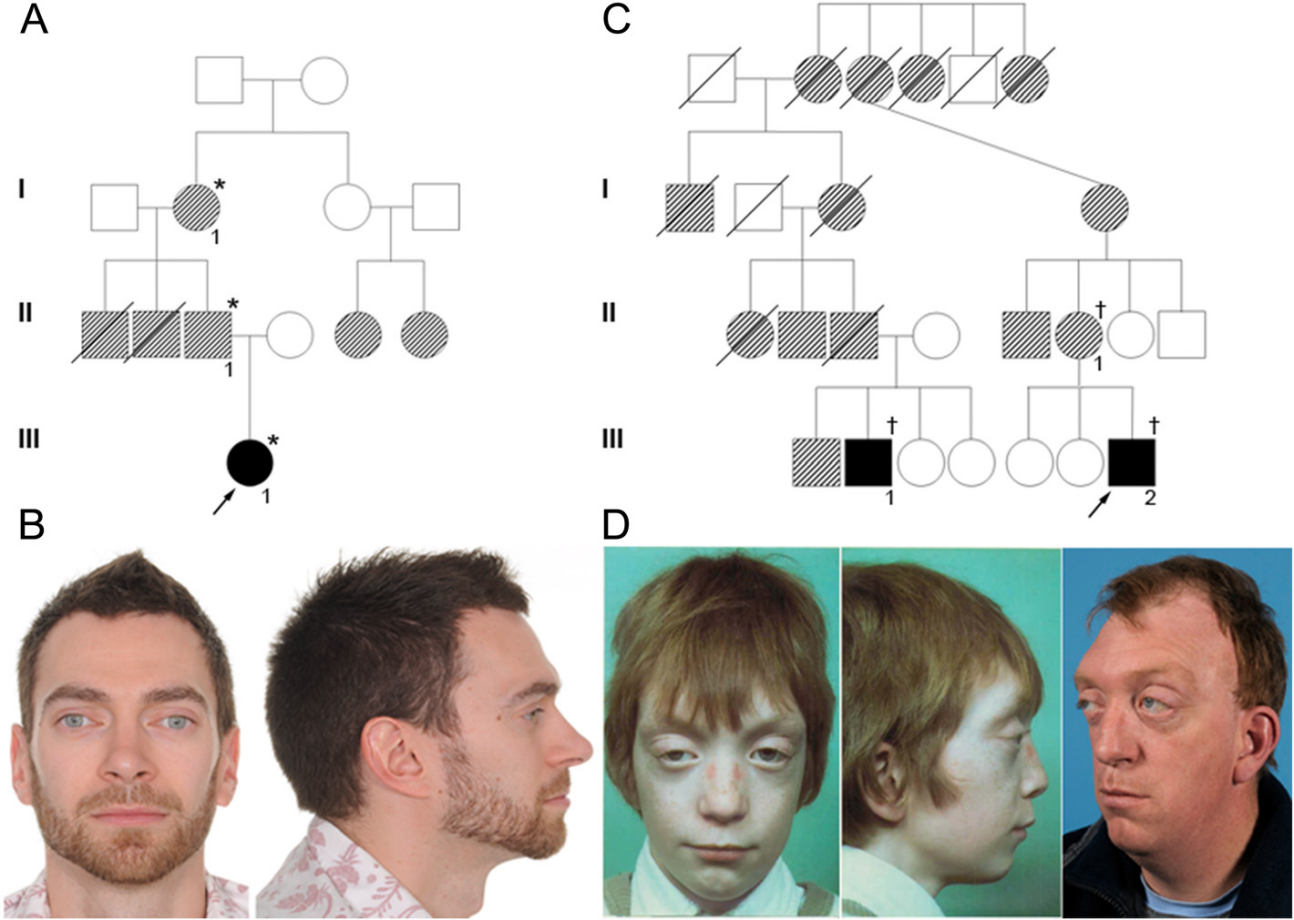
**Pedigrees and facial features of individuals with*FGFR2*mutations. A**, Pedigree of Family 1; solid symbols represent clinically confirmed craniosynostosis and hatched symbols represent individuals with a similar crouzonoid appearance but without confirmed craniosynostosis. *confirmed heterozygosity for c.1083A>G. **B**, Facial appearance of II-1 from Family 1 aged 33 years. **C**, Abbreviated pedigree of Family 2; notation of symbols as in part A.†confirmed heterozygosity for c.1083A>T. **D**, Facial appearance of III-1 from Family 2 aged 8.75 years (left) and 43 years (right).

Individual III-1 (Figure 1A) was born after a normal pregnancy and was referred for craniofacial assessment at two years of age because prominent eyes and a head tilt. Her father II-1 (Figure 1B), grandmother I-1 and two deceased uncles were all said to have a similar appearance with prominent staring eyes, but had not required any surgery. She had an occipitofrontal circumference (OFC) of 50 cm (+1.1 SD) and was noted to have a mildly crouzonoid appearance, with slight exorbitism and midface retrusion. In view of the mild crouzonoid features, sequencing of *FGFR2* exons IIIa and IIIc was requested. This demonstrated a heterozygous c.1083A>G [p.(Pro361Pro)] variant within exon IIIc, which was also present in II-1 and I-1.

Computed tomography (CT) of the skull of individual III-1 at the age of 2.8 years demonstrated right lambdoid and occipitomastoid synostosis, all other major cranial sutures being patent. Ophthalmological review identified slightly reduced visual acuity and a latent divergent squint with slight left hypophoria. The patient is now four years old and has not undergone any surgical intervention, as she has a good overall head shape with no major midface retrusion, is making good developmental progress, and has no features to suggest significant intracranial restriction.

### Family 2

The male proband (III-2 in Figure 1C), born after an uneventful pregnancy, was referred for craniofacial assessment at the age of three months. Physical examination showed a mild cloverleaf skull with temporal bulging and reduced OFC (36 cm; −2.2 SD), hypertelorism, and severe exorbitism mainly at the infra-orbital level. Skull X-ray and CT showed pansynostosis and multiple craniolacunae, with no intracerebral anomalies.

Owing to the severe peri-orbital features and the absence of deformations of the upper and lower extremities a clinical diagnosis of Crouzon syndrome was suggested. The patient’s mother, grandmother and several cousins were reported to show mild facial features also suggestive of this diagnosis.

The proband underwent fronto-orbital advancement at the age of five months. Since the occiput was still severely flattened and both lambdoid sutures were fused, occipital craniotomy and remodelling was performed at the age of twelve months. Clumsiness and motor delay were first noted aged 18 months; psychological testing at the age of 12.8 years gave scores for non-verbal intelligence of 80 (SON-R) and visual-motor integration of 81. Clonidine was prescribed due to high distractibility and he underwent special education. During childhood, the exorbitism increased requiring further orbital advancement and cranial vault remodelling at the age of eight years. Several deciduous and permanent teeth were extracted because of Class III malocclusion and dental crowding.

**Figure 2:**
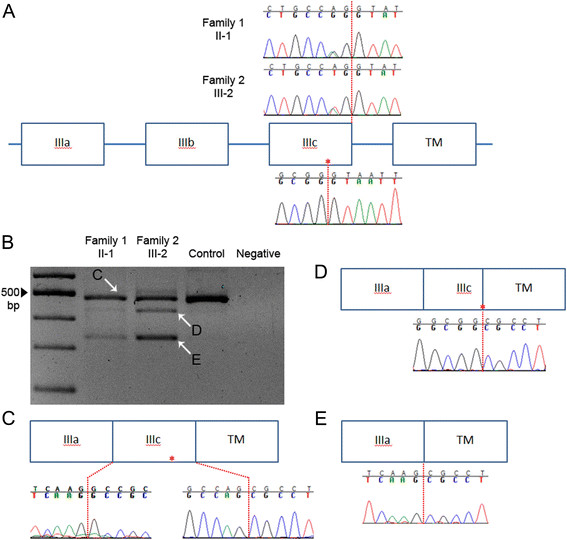
**Genomic context and consequences of*FGFR2*mutations. A**, Schematic representation of genomic region affected by c.1083A>G and c.1083A>T mutations (not to scale). IIIa, IIIb and IIIc denote exons of *FGFR2* encoding the 3^rd^ immunoglobulin-like domain; note that physiological skipping of exon IIIb normally occurs in blood mRNA.TM, exon encoding transmembrane domain. Sequencing of genomic DNA demonstrates heterozygosity for c.1083A>G (II-1, Family 1) or c.1083A>T (III-2, Family 2) mutation at the −2 position from the end of exon IIIc (indicated by dashed red line; the upper line in each trace shows the wild type sequence). Below the cartoon is shown the genomic sequence around the cryptic donor splice site within exon IIIc (marked with red asterisk). **B**, Amplified cDNA corresponding to c.1083A>G and c.1083A>T mutations (same individuals as for genomic analyses), demonstrates two additional smaller products (indicated with white arrows **D** and **E**), in addition to the normal **(C)** product. **C**, In the cDNA product coincident with the wild type band (II-1, Family 1), the mutant allele at the penultimate position of exon IIIc is not represented, indicating complete skipping of normal splicing. The consequences for splicing of the mutant allele (sequence traces illustrated are from III-1, Family 2) are either to activate the cryptic splice donor site within exon IIIc **(D)** or to skip exon IIIc completely **(E)**.

Genetic testing of *FGFR2* was performed, identifying the heterozygous point mutation c.1083A>T [p.(Pro361Pro); Figure 2A]. This variant was also present in his mother II-1.

Independently of these events, a second patient (III-1) was referred to the same clinic at the age of ten years with a scaphocephalic head shape. He had previously undergone vault remodelling at the age of 16 months owing to bicoronal synostosis. At the time of referral, he had an occipito-frontal circumference of 48.5 cm (−2.8 SD), hypertelorism and severe exorbitism. In addition, he had mild maxillary hypoplasia and both 2nd premolars of the lower jaw were absent. Ophthalmic examination showed myopia with divergent strabismus of the right eye associated with reduced visual acuity. A monobloc procedure without distraction (Le Fort III and an advancement of the forehead) was performed.

Due to the severe peri-orbital features a diagnosis of Crouzon syndrome was suggested. Analysis of *FGFR2* identified the heterozygous point mutation c.1083A>T [p.(Pro361Pro)]. His father, grandmother and great-grandmother had a similar craniofacial appearance. Based on the pedigree analysis, it is evident that III-1 and III-2 are third cousins and that the *FGFR2* mutation present in these two branches is identical by descent (Figure 1C). Apart from III-1 and III-2, none of the other affected family members had undergone craniofacial surgery.

## Materials and methods

Ethics approval for the study was obtained from NRES Committee London - Riverside (09/H0706/20) and the Medical Ethical Committee of the Erasmus University Medical Center Rotterdam (MEC-2013-547). Venous blood was collected into PAXgene Blood RNA tubes (Qiagen) from individuals II-1 (Family 1) and III-1 (Family 2), and RNA was extracted according to the associated protocol. cDNA was synthesized using the Fermentas RevertAid First-Strand Synthesis kit with random hexamer primers according to the manufacturer’s instructions.

cDNA was amplified using a forward primer in *FGFR2* exon IIIa (5′-TCGGAGGAGACGTAGAGTTTGTCTGC-3′) used in combination with a reverse primer in exon 11 (encoding the transmembrane (TM) domain; 5′-TGTTACCTGTCTCCGCAGGGGGATA-3′). DNA bands were cut out and gel purified using the Q-Spin gel extraction kit (Geneflow). Dideoxy sequencing was carried out on the resulting DNA products. The resulting cDNA products were numbered according to NCBI Reference Sequence: NM_000141.4.

## Results

The synonymous variants c.1083A>G and c.1083A>T occur at the −2 position of the 5′ (donor) splice site of *FGFR2* exon IIIc (Figure 2A). The neural network splice site predictor (http://www.fruitfly.org/seq_tools/splice.html) generates a score for the wild type donor of 0.88, which is reduced to 0.37 by the A>G transition, and to 0.19 by the A>T transversion. In these circumstances, use of a cryptic splice site (score 0.84) within exon IIIc, 51 nucleotides upstream from the end of the exon, is expected based on analysis of a previous mutation c.1084+3A>G [17]. This would lead to an in-frame deletion of 17 amino acids.

Amplification of cDNA from individuals heterozygous for either the *FGFR2* c.1083A>G or the c.1083A>T variants demonstrated the presence of two additional bands, not present in the wild type control, at approximately 430 bp and 330 bp (Figure 2B). Sequencing of the normal 479 bp product from these individuals showed complete absence of the mutant allele (illustrated in Figure 2C for II-1 from Family 1), indicating that both mutations abolish use of the normal exon IIIc donor splice site. Sequencing of the ~430 bp product confirmed that the cryptic splice donor within exon IIIc was preferred in the mutant allele (Figure 2D), while the ~330 bp product demonstrated complete skipping of exon IIIc (Figure 2E).

## Conclusions

In humans there is a consensus sequence around the 5′ splice donor site: A, C or T, AG / gtaagt (where / indicates the exon-intron boundary). Mutations of the consensus A at the penultimate nucleotide of the exon have been associated previously with loss of splicing at the donor site [18],[19]. Other mutations near the *FGFR2* exon IIIc splice donor site have also been described (Table 1), all associated with a mild Crouzon phenotype. It is likely that the p.(Ala362Ser) substitution [20] in fact exerts its pathogenic effect via the G>T transversion at the −1 position in the exon, rather than as a missense substitution, but this was not tested experimentally.

**Table 1:** **Summary of mutations affecting correct splicing of the** ***FGFR2*** **exon IIIc donor site**

**Mutation**	**Protein**	**Proposed effect**	**Experimental demonstration**	**Reference**
c.1032G>A	p.(Ala344Ala)	Activation of cryptic splice site	Yes	Reardon et al. 1994 [6]; Li et al. 1995 [21]; Del Gatto & Breathnach 1995 [22]
c.1083A>G	p.(Pro361Pro)	Loss of normal donor site with use of alternative cryptic splice site	Yes	This study
c.1083A>T	p.(Pro361Pro)	As above	Yes	This study
c.1084G>T	p.(Ala362Ser)	Annotated as missense but likely to affect splicing	No	Everett et al. 1999 [20]
c.1084+3A>G	-	Loss of normal donor site with use of alternative cryptic splice site	Yes	Kan et al. 2004 [17]
c.1084+3A>C	-	Loss of normal donor site with use of alternative cryptic splice site	No	Cornejo-Roldan, Roessler & Muenke 1999 [23]; Kress et al. 2000 [24]

In the cases reported here, the wild type splice donor site is abolished by the A>G or A>T mutations at the −2 position from the intron, leading to the cryptic site being preferred. The apparently greater amount of mutant cDNA products associated with the A>T mutation appears to correlate both with the greater predicted disruption of the splice site and with the more severe phenotype in clinically affected individuals from Family 2 compared with Family 1. However, since the A>G mutation did not support use of the normal exon IIIc donor splice site (Figure 2C), other explanations for these differences are possible, such as differing proportions of cell types in the blood samples analysed, and/or differences in genetic background.

Utilisation of the cryptic donor would lead to an in-frame deletion of the last 17 amino acids of exon IIIc (p.Gly345_Pro361del), including four residues that form specific contacts with the ligand FGF2 [25]. However as craniosynostosis-causing FGFR mutations function in a constitutively active dominant manner [26], it is also likely that in these individuals a mutant protein is formed which is prone to forming covalently-linked dimers [26], leading to variable features of Crouzon syndrome.

Our case reports document the range of phenotypic consequences associated with these particular mutations. Whilst a mild crouzonoid phenotype was generally evident, only a minority of individuals developed overt craniosynostosis requiring calvarial surgery. Orthodontic problems may also occur but these were not fully documented in our study. The causes of the clinical variability are unknown, although one potential factor may be the extent of intrauterine fetal head constraint [27].

In conclusion, mutations near the *FGFR2* exon IIIc splice sites should be carefully evaluated as to whether they may be pathogenic, even if they are synonymous or outside the canonical AG/GT splice acceptor/donor sequences. In particular, the mutations described here are associated with variable Crouzon syndrome features and affected families should be counselled as such.

## Consent

Written informed consent was obtained from the patients for publication of this Case report and any accompanying images. A copy of the written consent is available for review by the Editor of this journal.

## Competing interests

The authors declare that they have no competing interest.

## Authors’ contributions

Patient ascertainment and assessment: JACG, JR, AJMH, SAW, IMJM, AOMW. Experimental analysis: ALF, HL, AMWO. Supervised experiments: TL, AOMW. Drafted paper: ALF, JACG, AOMW. All authors approved the paper.
